# The challenges of treating eating disorders in Maori

**DOI:** 10.1186/2050-2974-3-S1-O32

**Published:** 2015-11-23

**Authors:** Zoe Williams, Kate De Bruyn, Miriama Scott

**Affiliations:** 1 CMDHB Auckland, New Zealand

## 

NZ is considered a bicultural society; Maori make up approx 15% of the population (NZ census 2013). Maori have strong cultural traditions and beliefs around food, with food not only determining physical health but also emotional, psychological and spiritual wellness (HRC report). Food is also strongly linked to customs and cultural values. The implications and understanding of eating disorders in this group are therefore complex.

Mental Health services traditionally struggle to both engage and treat the Maori population. Eating disorders rates are low overall in the general population; research suggesting that there was a very low prevalence overall for eating disorders in Te Rau Hinengaro. However, the highest prevalence of eating disorders, particularly bulimia, was in Māori (BPJ 2010).

With this in mind there are several challenges to consider when planning and offering services to treat eating disorders in this population.

The CMDHB covers an area with high Maori population and provides a culturally specific service for both adults and young people. The rates of referral of Maori to the CMDHB Eating Disorders Service are low overall, despite this there is a recognised unmet need in terms of recognition and support for these difficulties. Often young people present with complex needs with eating issues part of this presentation. Traditional treatments pose dilemmas when considering the cultural aspects of engagement and working with maori families; as well as the explanatory models used to think about eating issues. This presentation will consider these challenges and use a case example to support ideas to improve services and outcomes in this population.

